# The Effect of Caloric Restriction with and without n-3 PUFA Supplementation on Bone Turnover Markers in Blood of Subjects with Abdominal Obesity: A Randomized Placebo-Controlled Trial

**DOI:** 10.3390/nu13093096

**Published:** 2021-09-02

**Authors:** Urszula Razny, Joanna Goralska, Philip C. Calder, Anna Gruca, Caroline E. Childs, Maria Kapusta, Krystyna Slowinska-Solnica, Aldona Dembinska-Kiec, Bogdan Solnica, Malgorzata Malczewska-Malec

**Affiliations:** 1 Department of Clinical Biochemistry, Jagiellonian University Medical College, Skawinska 8, 31-066 Krakow, Poland; j.goralska@uj.edu.pl (J.G.); anna.gruca@uj.edu.pl (A.G.); maria.kapusta@uj.edu.pl (M.K.); krystyna.slowinska-solnica@uj.edu.pl (K.S.-S.); mbkiec@cyf-kr.edu.pl (A.D.-K.); bogdan.solnica@uj.edu.pl (B.S.); m.malczewska-malec@uj.edu.pl (M.M.-M.); 2 School of Human Development and Health, Faculty of Medicine, University of Southampton, Tremona Road, Southampton SO16 6YD, UK; P.C.Calder@soton.ac.uk (P.C.C.); C.E.Childs@soton.ac.uk (C.E.C.); 3 NIHR Southampton Biomedical Research Centre, University Hospital Southampton NHS Foundation Trust and University of Southampton, Tremona Road, Southampton SO16 6YD, UK

**Keywords:** bone turnover, obesity, caloric restriction, n-3 PUFAs

## Abstract

Weight loss contributes to an increased risk of hip fracture, especially in postmenopausal women. Omega-3 polyunsaturated fatty acid (n-3 PUFA) supplementation could diminish the adverse effect of weight loss on bone health. The aim of this randomized, placebo-controlled, double-blind parallel trial was to investigate the effect of caloric restriction and n-3 PUFA supplement intake on osteogenic markers (carboxylated osteocalcin (Gla-OC); procollagen I N-terminal propeptide (PINP)), as well as a bone resorption marker (C-terminal telopeptide of type I collagen (CTX-I)) in a serum of 64 middle aged individuals (BMI 25–40 kg/m^2^) with abdominal obesity. Bone remodeling, metabolic and inflammatory parameters and adipokines were determined before and after 3 months of an isocaloric diet (2300–2400 kcal/day) or a low-calorie diet (1200 kcal/day for women and 1500 kcal/day for men) along with n-3 PUFA (1.8 g/day) or placebo capsules. CTX-I and adiponectin concentrations were increased following 7% weight loss independently of supplement use. Changes in CTX-I were positively associated with changes in adiponectin level (rho = 0.25, *p* = 0.043). Thus, an increase in serum adiponectin caused by body weight loss could adversely affect bone health. N-3 PUFAs were without effect.

## 1. Introduction

Obesity is a disorder which is a major public health problem due to its associated complications and increased risk of chronic disease [1,2,3]. To prevent these adverse health impacts, weight reduction is a key therapeutic goal in people with obesity. Lower energy intake (caloric restriction) and changing lifestyle by increasing energy expenditure (physical activity) are the first treatment options and can be effective. Weight loss exerts beneficial effects on health by improving metabolic parameters (serum triglyceride concentration, glucose tolerance and insulin sensitivity) as well as decreasing blood inflammatory markers and cardiovascular risk factors [3]. However, some studies have documented that weight loss due to caloric restriction is associated with increased risk of hip fracture, especially in overweight and obese postmenopausal women [4,5,6]. It was reported that weight reduction resulted in an increase in blood markers of bone resorption, suggesting bone loss. However, the effect of caloric restriction on bone turnover in midadulthood is still unclear because some studies reported an increase in bone mass after intervention [7,8], while others report a decrease or no change [4,5,6].

In addition to caloric restriction, omega-3 polyunsaturated fatty acids (n-3 PUFAs) could reduce obesity-related complications and chronic disease risk through a reduction in inflammation [9,10,11,12,13]. Moreover, n-3 PUFA supplementation could beneficially alter bone metabolism by decreasing bone resorption and so help to maintain bone mass, as reported in cell model systems and animal studies [14,15]. It was also shown that n-3 PUFAs increase intestinal calcium absorption and enhance activation of osteoblastic activity by increasing procollagen and osteocalcin, which are markers of bone mineralization [16]. N-3 PUFAs were also reported to inhibit osteoclast activity [10,17] and bone resorption by downregulating macrophage colony stimulating factor (M-CSF), microphthalmia-associated transcription factor (MITF), and receptor activator for nuclear factor κ B (RANK) expression [18,19]. Some human studies showed that n-3 PUFA consumption prevented bone loss and reduced the risk of osteoporosis [20]. Experimental findings revealed that the effect of n-3 PUFAs is driven by their lipid mediators such as resolvin E1 (RvE1) which is produced from eicosapentaenoic acid (EPA). RvE1 was reported to inhibit osteoclastogenesis [21] and upregulate osteoprotegrin (OPG), restore the receptor activator for nuclear factor κ B ligand (RANKL)/OPG ratio and attenuate RANKL-induced osteoclastic activity [22]. Data shown by Zalloua et al. indicated that enhanced consumption of seafood, a source of n-3 PUFAs, was associated with increased bone mineral density [23]. However, intervention trials with n-3 PUFA supplements in humans are limited and the findings are not clear. Moreover, there is only one study that has focused on the effect of caloric restriction with or without n-3 PUFA supplementation in young adults [24].

This study aimed to investigate the effect of caloric restriction (low calorie versus isocaloric diet) and n-3 PUFA supplement intake (n-3 PUFA versus placebo) lasting for three months on osteogenic markers ((carboxylated osteocalcin (Gla-OC) and procollagen I N-terminal propeptide (PINP)) as well as a bone resorption marker (C-terminal telopeptide of type I collagen (CTX-I)) in blood of middle-aged adults with abdominal obesity. This is the first trial in which the effect of caloric restriction with and without n-3 PUFA supplementation on bone turnover in humans is investigated. We hypothesize that 3 months of n-3 PUFA supplementation in subjects on an isocaloric diet result in an increase in bone formation markers, that a short-term low-calorie diet increases the bone resorption marker, and that n-3 PUFA supplementation will mitigates the negative effect of caloric restriction on the bone resorption marker.

## 2. Materials and Methods

### 2.1. Participants

Subjects with overweight or obesity (BMI 25−40 kg/m^2^) with abdominal obesity (waist circumference >102 cm in men and >88 cm in women) aged 25−65 years were eligible to participate in the trial examining the differences in serum bone turnover markers across groups as the primary outcome. Sample size was calculated as 15 per arm to detect a 40% change in bone turnover markers at a *p* value < 0.05 with a power of 80%. To estimate sample size CTX-I was used.

Subjects with diabetes, other endocrine disorders, chronic inflammatory diseases and kidney or liver dysfunction were excluded. Subjects participating in the study had not taken lipid-lowering or anti-inflammatory drugs or supplements containing vitamins A, D, C or E, β-carotene or PUFAs. Fish consumption was not allowed during the study period.

Participants were recruited from the Out-Patient Clinic of Obesity and Lipid Disorders and the Department of Metabolic Disorders, Jagiellonian University Medical College, Krakow, Poland.

### 2.2. Study Design and Intervention

We performed a 3 month randomized, double-blind, and placebo-controlled parallel and single-center trial. The trial was performed in accordance with the Code of Ethics of the World Medical Association (Declaration of Helsinki) and with the Good Clinical Practice guidelines. Ethical approval was provided by the Bioethics Committee of the Jagiellonian University in Krakow, Poland (written consent, decision number KBET/82/B/2009), and all subjects gave written informed consent prior to participation in the study. The trial was conducted at the Department of Clinical Biochemistry, Jagiellonian University, Medical College, Krakow, Poland. The trial was registered at isrctn.com (accessed on 20 February 2015) as ISRCTN11445521. The recruitment of trial participants began in September 2009 and was completed in July 2013.

Women and men were screened for eligibility at their first medical visit. All recruited subjects had detailed medical examination and standard laboratory exams conducted to evaluate their health status and to exclude volunteers who did not meet inclusion criteria. All subjects had their height and weight measured, and BMI was calculated. Subjects who fulfilled inclusion criteria were provided with study information and, before giving written consent to participate, were counselled by a research assistant.

Recruited subjects were randomized using an independent online computerized randomization system by a member of administrative staff who was not involved in recruitment, clinical care or dietary advice. Randomization was conducted at the level of the individual and was stratified by sex and age. Before randomization, recruited subjects underwent an adaptation period of two weeks. During this period of time, participants were asked by a nutritionist to adopt a diet containing the amount of calories according to individual caloric requirement, which was calculated taking into account the resting metabolism and the level of physical activity for each subject. This isocaloric diet (2300−2400 kcal/day) contained 57% of energy from carbohydrates, 30% from fat and 13% from protein. Subjects were advised by a dietician on how to apply this diet and were given written instructions. After the adaptation period, each participant had a dietary consultation and was randomly assigned to one of the four groups.

Subjects, who were randomized to the first or second group, were advised by a dietician to adopt the isocaloric diet (2300−2400 kcal/day) and to consume 3 times per day capsules after a meal (IS + placebo group, IS + n-3 PUFA group) whereas those assigned to the third or fourth group were advised to adopt a low-calorie diet and to consume 3 times per day capsules after a meal (CR + placebo group, CR + n-3 PUFA group, respectively). The caloric value of the low-calorie diet (CR) was 1200 kcal/day for women and 1500 kcal/day for men, and 60% of energy was provided from carbohydrates (with low glycemic index), 15% from protein and 25% from polyunsaturated fat. During the implementation of the 3 month intervention diet, all subjects were under the supervision of a dietitian with visits every two weeks. During these visits, a dietary questionnaire was completed and, additionally, a 24 h dietary interview. In order to assess the dietary compliance and the intake of essential nutrients and vitamins, each subject was asked to complete a 3 day food diary before and after the 3 month diet intervention. Each subject had the possibility of contact by phone with a dietitian to clarify any concerns. The amounts of calories, nutrients and vitamins consumed were calculated using the “Dietician” program prepared by the Food and Nutrition Institute in Warsaw (Poland).

Placebo (corn oil) or n-3 PUFA (EPAX 1050 TG; EPAX, Alesund, Norway) capsules were identical in size, shape and appearance and contained 4 mg of vitamin E per capsule. Three n-3 PUFA capsules containing 1.8 g docosahexaenoic acid (DHA) + eicosapentaenoic acid (EPA) in a ratio of 5:1. 1.8 g of n-3 PUFAs were chosen because such an amount could be delivered in the human diet, since it is equivalent to 4 servings of oily fish per week. Subjects were advised to swallow their capsules immediately after a meal. Due to similar appearance of the placebo and n-3 PUFA capsules, blinding of participants and nutritionists was possible. Considering the nature of the trial, the dietician and participants could not be blinded in terms of caloric content of diets consumed for 3 months. However, where possible, subjects, medical staff, laboratory technicians, data analysts and outcome assessors and study coordinators were blinded to treatment allocation.

### 2.3. Assessment of Compliance

Compliance to the supplements was assessed by counting returned capsules. Additionally, fatty acid composition of plasma phosphatidylcholine (PC) and of erythrocyte membranes was measured before and after intervention. A cut-off of a 20% increase in “omega-3 index”, which is the sum of EPA and DHA in erythrocyte membranes, was used to identify compliers in the n-3 PUFA group and noncompliers in the placebo group.

### 2.4. Outcome Measures

Primary outcome measures of the trial were changed in bone turnover markers between baseline (after two weeks of isocaloric diet adaptation period) and 3 months of dietary intervention. After two weeks of the adaptation period (baseline) and at the end of the 3 month intervention period, venous blood was collected after a 12 h overnight fast and was used for measurement of the following bone turnover markers: Gla-OC, undercarboxylated osteocalcin (Glu-OC), CTX-I, osteopontin, PINP. Serum Gla-OC and Glu-OC were determined by ELISA (Takara, Japan). Intra- and interassay coefficients of variation were: <4.8% and <2.4% (Gla-OC) and <6.66%, and <9.87% (Glu-OC), respectively. Total osteocalcin level was calculated as the sum of Gla-OC and Glu-OC. Serum CTX-I was measured by ELISA (BioVendor, Asheville, NC, USA). Intra- and interassay coefficients of variation were: <2% and <7%. Serum PINP was measured by ELISA (Cloud-Clone Corp., Katy, TX, USA). Intra- and interassay coefficients of variation were: <10% and <12%

#### 2.4.1. Anthropometric Measures

At baseline (after two weeks of adaptation period) and at the end of 3 months intervention, anthropometric measurements (BMI, hip and waist circumference and adipose tissue content) were made. Body composition changes including fat content, muscle mass and water content were measured by Tanita Body Composition Analyser BC-418 (Tanita, Tokyo, Japan).

#### 2.4.2. Secondary Blood Outcome Measures

Before and after the intervention, after a 12 h overnight fast venous blood was collected for measurement of serum glucose, insulin, glucose-dependent insulinotropic poly-peptide (GIP), nonesterified fatty acids (NEFAs), total cholesterol, HDL-cholesterol and triglycerides, adipokines (leptin, adiponectin, resistin, visfatin), adhesion molecules (sE-Selectin, soluble vascular cell adhesion protein 1 (sVCAM-1), soluble platelet endothelial cell adhesion molecule 1 (sPECAM-1)), and proinflammatory markers (C-reactive protein (CRP), interleukin 6 (IL-6) and monocyte chemoattractant protein 1 (MCP-1)). Serum glucose was measured using glucose oxidase method (ELITech Clinical Systems, Sées, France). Intra-assay and inter-assay coefficients of variation were 2.3% and 3.5%, respectively. Serum triglycerides were determined by triglycerides oxidase method (ELITech Clinical Systems, France). Within-day and between-day precision CVs were 1.4% and 3.4%, respectively. Insulin was determined by an immunoradiometric method (INS IRMA DIAsource, ImmunoAssays, Ottignies-Louvain-la-Neuve, Belgium). The within-run and between-run CVs were 2.1% and 6.5% respectively. Basal insulin resistance was estimated using homeostasis model of assessment (HOMA-IR) [25]. GIP was measured using ELISA (EMD Millipore, St Charles, MO, USA). Within-run CV was 6.1% and between-run CV was 8.8%. The limit of quantitation was 8.2 pg/mL. NEFAs were measured using an enzymatic colorimetric method. Serum total cholesterol was determined by the cholesterol oxidase method (ELITech Clinical Systems, France). Within-run CV was 1.4% and between-run CV was 3.8%. HDL-cholesterol was determined by an enzymatic colorimetric method (ELITech Clinical Systems, France). Within-run and between-run CVs were 2.1% and 2.8%, respectively. LDL-cholesterol was calculated using the Friedewald formula. Serum leptin, adiponectin (adipocyte complement-related protein of 30kDa, Acrp 30), resistin, IL-6, sE-Selectin, MCP-1, sVCAM-1, osteopontin and fibroblast growth factor 21 (FGF-21) were assayed using ELISA (R&D Systems Europe, Abingdon, United Kingdom). Within- and between-run imprecision CVs were 3% and 4% (leptin), 4% and 6% (adiponectin), 5.3% and 8.2% (resistin), 6% and 7% (IL-6), 6% and 8% (sE-Selectin), 5% and 6% (MCP-1), 3.5% and 7.7% (sVCAM-1), 4.0% and 6.6% (Osteopontin), and 3.9% and 10.9% (FGF-21). Vitamin D was measured by electrochemiluminescence on Cobas PRO analyzer (Roche Diagnostics, Mannheim, Germany). NEFAs concentration was measured immediately in nonfrozen plasma by enzymatic quantitative colorimetric method (Roche Diagnostics GmbH, Germany).

Venous blood was also collected for measurement of plasma phosphatidylcholine (PC) fatty acid composition and fatty acid content of erythrocyte membranes at baseline and at the end of the trial. The fatty acid composition of plasma PC and red blood cells (RBC) was assessed by gas chromatography using methods described previously [26]. Chloroform/methanol (2:1 vol/vol) was used to extract total lipid from plasma or erythrocytes. PC was separated by solid phase extraction on Bond Elut cartridges (Varian, Palo Alto, CA, USA). Plasma PC and erythrocyte lipids were saponified: in the presence of sulphuric acid containing 2% methanol, fatty acid methyl esters were formed by heating at 50 °C for 2 h, extracted into hexane and then concentrated by evaporation under nitrogen. Separation of fatty acid methyl esters was performed by gas chromatography on a Hewlett Packard 6890 gas chromatograph (Hewlett Packard, CA, USA) fitted with a BPX70 column (SGE Europe, Milton Keynes, Bucks, UK). Run conditions are described elsewhere [26]. Identification of fatty acids was by comparison of their retention times with those of authentic standards. Data are presented as percentage contribution to the total fatty acid pool.

### 2.5. Data Analysis

Nominal data were analyzed by chi-square (χ^2^) test. The Shapiro−Wilk test was used to analyze data for a Gaussian distribution and Levene’s test was used to verify the homogeneity of variance. Continuous variables were transformed if required. Normally distributed data are presented as mean ± SD, otherwise as median and lower and upper quartile range (25, 75%). Differences between the four studied groups were analyzed by one-way ANOVA or Kruskal−Wallis and Dunn test (for non-normally distributed data). Potential differences in biochemical measurements between baseline and after intervention were calculated with repeated measures by ANOVA and Tukey post hoc test. The Spearman rank correlation was used to find an association between non-normally distributed data. *p* values less than 0.05 were considered significant. Statistical analyses were performed with the Statistica software (StatSoft).

## 3. Results

**Figure 1:**
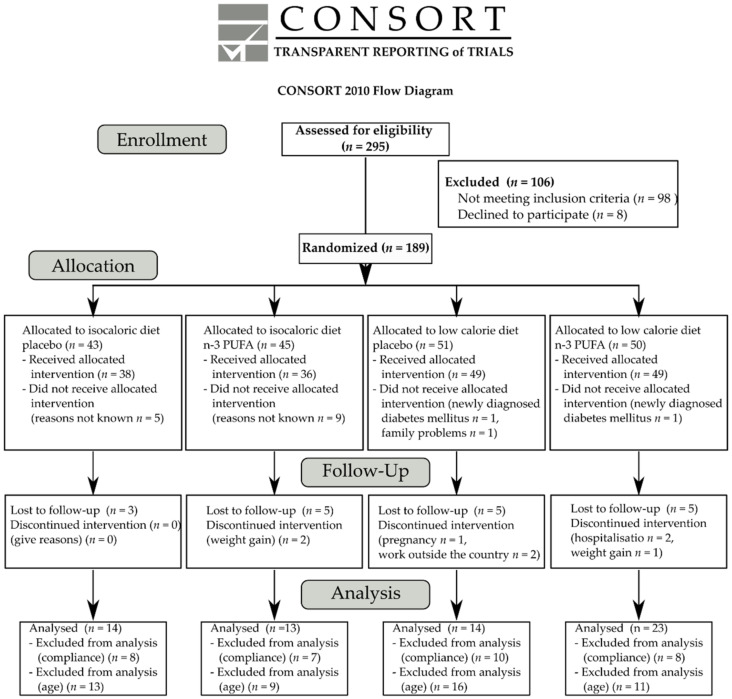
CONSORT flow diagram.

The aim of the trial was to investigate the effect of n-3 PUFA supplementation as well as a moderate low-calorie diet (1200 kcal/day for women and 1500 kcal/day for men) for three months on bone turnover markers in subjects with abdominal obesity. The recruitment of study participants began in September 2009 and was completed in July 2013. Of the 295 volunteers assessed for eligibility, 98 were excluded because of not meeting inclusion criteria and a further eight volunteers declined to participate. Thus, 189 subjects were enrolled in the study and randomly assigned to one of four intervention groups: isocaloric diet with placebo capsules (*n* = 43), isocaloric diet with n-3 PUFA capsules (*n* = 45), low-calorie diet with placebo capsules (*n* = 51), and low-calorie diet with n-3 PUFA capsules (*n* = 50). The progression of subjects through the study is shown in Figure 1. Of the 43 subjects assigned to isocaloric diet and placebo supplementation, five did not receive the intervention for reasons not known. In the second group, 36 participants received the allocated intervention, while nine subjects declined to participate after allocation. In turn, two subjects did not receive low-calorie diet and placebo supplementation one due to newly diagnosed diabetes mellitus type 2, and the other declined to participate because of family trouble. In the fourth group, 49 subjects received the low-calorie diet and n-3 PUFA capsules; one subject was excluded due to newly diagnosed diabetes mellitus type 2. After treatment allocation, eighteen subjects were lost to follow up (three subjects from first group, five subjects from isocaloric diet with n-3 PUFA capsules, six subjects from low-calorie diet with placebo capsules and four subjects from the fourth group). Eight subjects discontinued intervention: two subjects from isocaloric diet with n-3 PUFA capsules due to claiming that taking capsules leads to weight gain, three participants of the third group (one discontinued due to pregnancy, two subjects found new work outside the country), three subjects from the fourth group (one subject developed pneumonia that required hospitalization, and one subject required hospitalization for decompensation of preexisting heart failure, and one subject claimed that taking capsules leads to weight gain). The n-3 PUFA capsules were safe and well tolerated. No adverse effects were observed after DHA/EPA (5:1) supplementation.

Finally, 146 subjects completed the trial (35 from isocaloric diet with placebo, 29 from isocaloric diet with n-3 PUFA capsules, 40 from low-calorie diet with placebo capsules and 42 from low-calorie diet with n-3 PUFA capsules). Thirty-three subjects were excluded from analysis due to not meeting compliance criteria: 18 subjects from the placebo group (omega-3 index after supplementation increased more than 20%) and 15 subjects from the n-3 PUFA group (the increase in omega-3 index was less than 20% after supplementation). In addition, in order to exclude effects of age on bone remodeling markers, women with age above 45 years were excluded from analysis. Thus, full analyses were performed in 64 subjects: 14 in the isocaloric diet with placebo group, 13 in the isocaloric diet with n-3 PUFA group, 14 in the low-calorie with placebo group and 23 in the low-calorie with n-3 PUFA group. 

Baseline characteristics did not differ between the four studied groups of subjects (Table 1). The majority of the 64 participants included in the analysis were females (*n* = 35) aged 25–44 years, but the four groups did not differ in terms of age and sex (Table 1). Baseline concentrations of EPA and DHA in plasma PC as well as in red blood cell membranes were also similar among all studied groups at baseline (*p* > 0.05). Among the 64 participants of the study, 24% were overweight (*n* = 15), 50% (*n* = 32) had BMI between 30 and 34.99 kg/m^2^, and 26% (*n* = 17) presented second degree obesity. Subjects allocated to caloric restriction with placebo capsules had significantly higher BMI than those on the isocaloric diet with n-3 PUFA capsules (35.26 ± 3.14 versus 30.84 ± 3.48 kg/m^2^, *p* = 0.007). However, the studied groups did not differ with regards to waist circumference and adipose tissue mass. All subjects had abdominal obesity, because in women waist circumference was above 80 cm, whereas in men it was above 94 cm. Subjects allocated to the four intervention groups did not differ in terms of parameters of glucose and lipid metabolism. In 64% (*n* = 41) of trial participants, LDL-concentration exceeded the value of 3 mmol/L; 34 subjects had blood total cholesterol above 5 mmol/L, and 23 presented abnormal triglycerides (>1.7 mmol/L). With regard to parameters of glucose metabolism, 14 subjects had impaired fasting glucose (5.6–6.9 mmol/L), and in 66% of the participants, the HOMA-IR index exceeded 2.5 which may indicate presence of insulin resistance. The four groups did not differ with regards to baseline adipokines (leptin, adiponectin, resistin, visfatin and FGF-21). The blood concentration of proinflammatory markers (IL-6, MCP-1 and CRP) as well as cell adhesion molecules (sE-selectin, sVCAM-1 and sPECAM-1) was similar in all studied groups. There were also no differences in baseline serum concentration of vitamin D or bone turnover markers (osteopontin, PINP, CTX-I, Gla-OC and Glu-OC) among the four groups. All subjects had total blood vitamin D concentration below the reference range (<30 ng/mL).

**Figure 2:**
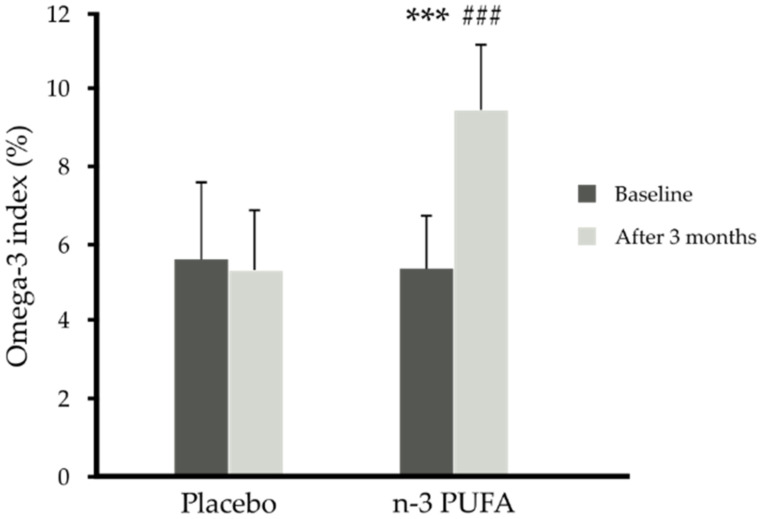
Omega 3 index (weight%) before and after 3 months of n-3 PUFA (*n* = 26) or placebo (*n* = 28) supplementation independent of diet. Data are shown as mean ± SD. Statistics: analyses were performed with repeated measures by ANOVA and Tukey post hoc test; significance: *** *p* < 0.001 after versus before n-3 PUFA supplementation, ### *p* < 0.001 after n-3 PUFA supplementation versus after placebo supplementation.

**Figure 3:**
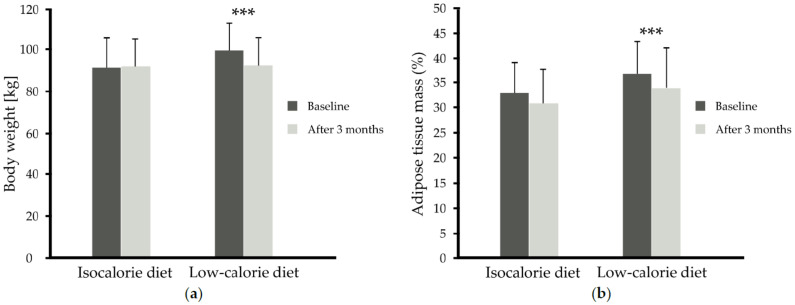
(**a**) Body weight (kg) before and after 3 months of isocaloric diet (*n* = 27) intervention or before and after 3 months of low-calorie diet (*n* = 37) intervention independent of supplementation, (**b**) adipose tissue mass (%) before and after 3 months of isocaloric diet (*n* = 27) intervention or before and after 3 months of low-calorie diet (*n* = 37) intervention independent of supplementation data are shown as mean ± SD. Statistics: analyses were performed with repeated measures by ANOVA and Tukey post hoc test; significance: *** *p* < 0.001 after versus before low-calorie diet.

In subjects taking 1.8 g/day n-3 PUFAs, plasma and red blood cell levels of EPA and DHA increased significantly (versus placebo, *p* < 0.001) independently of the diet used (Table 2, Figure 2). In turn, the low-calorie diet used for 3 months, independently of n-3 PUFA supplementation, resulted in weight loss by 7% (92.28 ± 13.49 versus 99.40 ± 13.35 kg) and adipose tissue mass loss (34.09 ± 7.90 versus 36.81 ± 6.44 %) in comparison to the isocaloric diet (*p* < 0.001, *p* = 0.05, respectively) (Table 2, Figure 2 and Figure 3a,b).

**Figure 4:**
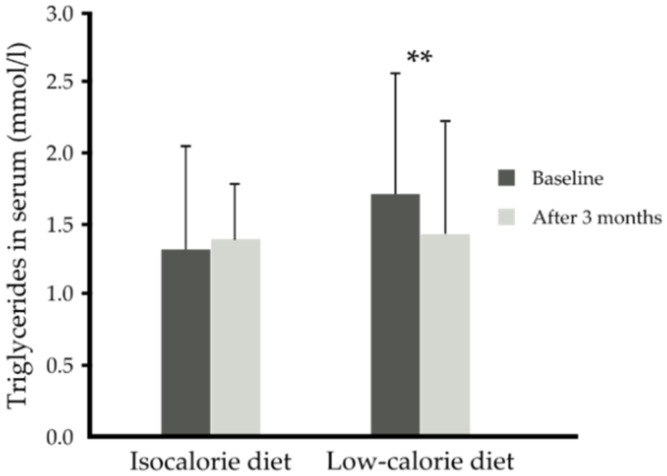
Serum triglycerides (mmol/L) before and after 3 months of isocaloric diet (*n* = 27) intervention or before and after 3 months of low-calorie diet (*n* = 37) intervention independent of supplementation. Data are shown as mean ± SD. Significance: ** *p* < 0.01 after versus before low-calorie diet.

With regards to the lipid and glucose metabolism parameters, the low-calorie diet independent of supplementation (placebo or n-3 PUFA) lowered serum triglycerides by about 16% (1.42 ± 0.81 compared to baseline 1.71 ± 0.86 mmoL/L) compared to the isocaloric diet (1.39 ± 0.72 versus 1.31 ± 0.73) (*p* = 0.003) (Table 3, Figure 4). However, neither supplementation nor diet led to the improvement of serum NEFAs, HDL-, LDL- and total cholesterol concentration (Table 3). There was a tendency for decreased serum glucose with the low-calorie diet (*p* = 0.054) or independently with n-3 PUFA supplementation (*p* = 0.060). The low-calorie diet independent of supplementation (placebo or n-3 PUFA) tended to lower insulin resistance, i.e., decreased HOMA-IR index (*p* = 0.056). However, neither supplementation nor diet influenced fasting GIP and insulin serum concentration (Table 3). 

**Figure 5:**
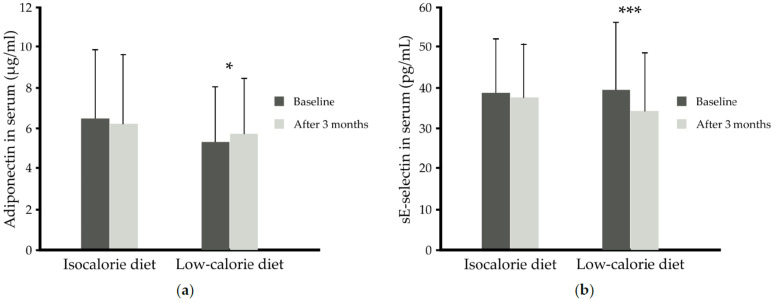
(**a**) Serum adiponectin (µg/mL) before and after 3 months of isocaloric diet (*n* = 27) intervention or before and after 3 months of low-calorie diet (*n* = 37) intervention independent of supplementation, (**b**) Serum sE-selectin (pg/mL) before and after 3 months of isocaloric diet (*n* = 27) intervention or before and after 3 months of low-calorie diet (*n* = 37) intervention independent of supplementation. Data are shown as means ± SD. Statistics: analyses were performed with repeated measures by ANOVA and Tukey post hoc test; significance: * *p* < 0.05 after versus before low-calorie diet, *** *p* < 0.001 after versus before low-calorie diet.

Unexpectedly, although the 3 month low-calorie diet decreased BMI, there was only a tendency for lower serum leptin concentration (*p* = 0.054), but independently of the supplement used, the low-calorie diet increased serum adiponectin (5.70 ± 2.75 versus 5.33 ± 2.71 µg/mL) (*p* = 0.008); in subjects on the isocaloric diet, the serum adiponectin concentration remained unchanged in comparison to baseline values (6.19 ± 3.39 versus 6.47 ± 3.38 µg/mL, *p* > 0.05) (Table 4, Figure 5a). The concentrations of other adipokines (resistin and visfatin) were similar in all four groups after intervention in comparison to baseline values.

With regards to proinflammatory markers, IL-6, MCP-1 and CRP were not significantly changed by either the low-calorie diet or the n-3 PUFAs. However, caloric restriction independent of supplementation led to lowering of the sE-selectin by about 13% (34.07 ± 14.43 versus 39.33 ± 16.70 ng/mL; *p* = 0.010) (Table 4, Figure 5b). The blood concentrations of other measured adhesion molecules such as sPECAM-1 and sVCAM-1 remained unchanged.

**Figure 6:**
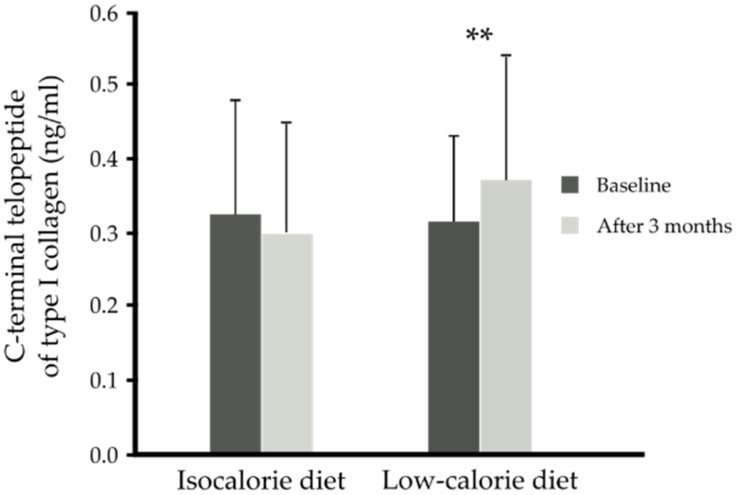
Serum C-terminal telopeptide of type I collagen (CTX-I) (ng/mL) before and after 3 months of isocaloric diet (*n* = 27) intervention or before and after 3 months of low-calorie diet (*n* = 37) intervention independent of supplementation. Data are shown as means ± SD. Statistics: analyses were performed with repeated measures by ANOVA and Tukey post hoc test; significance: ** *p* < 0.01 after versus before low-calorie diet.

Neither the low-calorie diet nor n-3 PUFA supplementation influenced the concentration of blood vitamin D, osteopontin, FGF-21 and bone formation markers (PINP, Gla-OC, Glu-OC). Three months isocaloric diet did not change blood CTX-I. However, 3 months caloric restriction independent of supplementation (placebo or n-3 PUFAs) resulted in an increase in the bone resorption marker CTX-I (0.37 ± 0.17 compared to 0.31 ± 0.12 ng/mL, *p* < 0.001) (Table 5, Figure 6). 

Moreover, changes in CTX-I concentration were inversely associated with changes in BMI (rho = −0.503, *p* < 0.001) and leptin concentration (rho = −0.262, *p* = 0.036). Analyses of correlations also revealed that changes in CTX-I positively correlated with changes in serum adiponectin level (rho = 0.253, *p* = 0.043) (Table 6). 

## 4. Discussion

The findings of this trial show that moderate energy restriction (greater than 5.0 MJ per day) [24] for a short period of time (3 months) can result in weight loss of 7% and also affects bone turnover, as indicated by higher serum concentration of a marker of bone resorption. It is well known that weight loss is associated with an increased risk of hip fracture in older individuals [27]. In premenopausal women, some studies, but not all, reported increased bone loss after weight reduction [28,29]. The current study investigated fairly short-term (3 months) moderate caloric restriction in middle aged men and women with obesity, and assessed blood biomarkers of bone turnover (osteocalcin, PINP and CTX-I), it did not assess bone mineral density. The novelty of the trial was comparison of the effect of weight loss by moderate caloric restriction to the control group of obese subjects who followed an isocaloric diet for 3 months. 

In agreement with previous studies [7,24], 3 month caloric restriction resulted in a statistically significant increase in blood concentration of the bone resorption marker, CTX-I. As reported previously, weight loss did not change the concentration of the bone formation marker PINP in serum [30]. There was also no effect on osteocalcin, carboxylated or undercarboxylated. Existing studies indicate, however, that 3 month moderate caloric restriction, contrary to very low or low-caloric restriction, results in an increase blood osteocalcin concentration [30]. However, on the other hand studies by Centi et al. indicated that caloric restriction (400 kcal/day) supplemented with 1000 mg/day calcium carbonate, 200 IU/day Vitamin D3, and 80 μg/day Vitamin K (phylloquinone) for 20 weeks did not lead to a change in serum total osteocalcin or undercarboxylated osteocalcin or the percentage of undercarboxylated osteocalcin in obese postmenopausal women [31]. Besides osteocalcin, we also investigated the effect of weight loss on another protein participating in energy metabolism and regulating bone mass, FGF-21. It was documented previously that FGF-21 is negatively correlated with bone mineral density at the spine [32]. However, in the current trial, weight loss did not result in a change in serum FGF-21. Similar to our study, Headland et al. also reported no change in blood FGF-21 concentration in individuals following an energy restriction diet (of 1000 kcal/day for women or 1300 kcal/day for men) for 12 months [33]. In the present study, there was also no change in serum osteopontin, a multifunctional protein which is distributed in many tissues and fluids. It is secreted by adipose tissue and is also produced by osteoblasts and osteoclasts. It participates in matrix remodeling and tissue calcification, monocyte/macrophage migration and chemotaxis, production of proinflammatory cytokines and chemokines [34]. It was reported that the serum concentration of osteopontin positively correlated with fat percentage [35]. In support of this relationship, it was demonstrated that 10% weight loss induced by caloric restriction resulted in a significant decrease in serum osteopontin [35]. The results of the present trial show that weight loss caused by short-term moderate caloric restriction contributes to changes in bone remodeling as indicated by increasing the serum level of CTX-I, which suggests a catabolic state of bone. Thus, diet-induced weight loss could exert adverse effects on bone health. Indeed, caloric restriction lasting for more than 6 months could lead to a decrease in total hip bone mineral density, because the complete cycle of bone remodeling takes 4−6 months [36]. Therefore, changes in blood bone turnover markers could indicate impairments in bone remodeling in obese individuals during weight reduction before the bone loss estimated by DXA becomes apparent [37]. In contrast to the effects on bone turnover, as documented previously, weight loss caused by caloric restriction exerts beneficial effects on metabolic parameters and prevents cardiovascular complications. In the current trial, 7% weight loss led to a decrease in blood triglycerides as well as an increase in serum adiponectin concentration in middle-aged adults with abdominal obesity. Moderate caloric restriction also tended to lower serum glucose concentration and insulin resistance. Serum concentration of the incretin GIP remained unchanged after caloric restriction, which is in agreement with a previous study in which weight loss of 6–8% did not affect this incretin [38]. In the current trial obese subjects did not respond to moderate caloric restriction with decreasing proinflammatory markers such as CRP, IL-6, MCP-1 or cell adhesion molecules (sVCAM-1, sPECAM-1). However, caloric restriction did lower sE-selectin. Consistent with most of the current findings, some previous trials did not document any changes in inflammatory cytokines after caloric restriction [39,40]. The effect of caloric restriction on proinflammatory markers appears to depend on the percentage of weight loss. Sola et al. [40] reported that a low-calorie diet (1200–1500 kcal/day for 2 months) did not contribute to lowering inflammation in obese subjects. Moreover, Magkos et al. [41] found that a modest weight loss of 5% improved only some metabolic parameters such as insulin sensitivity whereas a larger weight loss (16%) downregulated inflammatory gene expression in peripheral blood mononuclear cells of obese subjects [42]. In that study caloric restriction downregulated IL-6 mRNA and IL-1B expression and improved insulin sensitivity [42] as well as regulating gene expression in adipose tissue by increasing mitochondrial function [43]. It was reported previously that a modest weight loss of 10% reduces blood inflammatory markers and cardiovascular risk factors and increases serum adiponectin concentration [44,45]. Thus, our results are in general agreement with those documented by Sola and Magkos [40,41] taking into account the fact that in the trial subjects used moderate caloric restriction (1200 kcal/day for women and 1500 kcal/day for men) for 3 months, with a resulting weight loss of 7%. In our study, weight loss resulted in a tendency to lower leptin and insulin resistance. However, caloric restriction did not influence serum concentration of other adipokines such as visfatin and resistin. In a study by Greco et al. [46], caloric restriction (1200–1600 kcal/day) for 4 months also did not lead to changes in these two adipokines. 

Considering the beneficial effect of weight loss on health, further studies should be focused on the interventions or factors that could prevent bone loss. Randomized controlled trials indicate that changes in bone health caused by weight loss could be attenuated by calcium supplementation [8,47], higher dietary protein intake or increased physical activity [48]. Some studies performed in rat or cell models suggest that n-3 PUFAs stimulate calcium absorption and beneficially alter bone metabolism [16,49,50]. Additionally, n-3 PUFAs decrease tumor necrosis factor α (TNF-α) which is involved in bone destruction in rheumatoid arthritis and can also reduce the secretion of prostaglandin E2 (a potent stimulator of bone resorption) [51]. Thus, the current study investigated if addition of n-3 PUFAs in a dose of 1.8 g per day to an isocaloric diet or a moderately caloric-restricted diet for 3 months influenced bone turnover markers in subjects with abdominal obesity. Our study is the first to investigate the effect of n-3 PUFA supplementation on bone turnover markers in middle-aged subjects with abdominal obesity during caloric restriction diet in comparison to isocaloric diet and placebo. There is one study which studied the combination of moderate energy restriction and increased seafood consumption or n-3 PUFA capsules on bone metabolism in overweight young adults. Lucey et al.’s [24] study reported that neither fish oil capsules (3 g/day) nor whole fish (cod or salmon (3 × 150 g/day)) for 8 weeks had an effect on the increase in bone resorption markers induced by moderate energy restriction.

In our study, supplementation with n-3 PUFAs also did not significantly change serum concentration of bone formation markers (PINP, osteocalcin), indicating a lack of the anticipated effect. Moreover, adding n-3 PUFAs to a caloric-restricted diet did not mitigate the negative effect of weight loss on bone resorption. The response of bone turnover markers to n-3 PUFA supplementation appears to depend on the dosage and duration of supplementation. Similar to the current study, Dong did not observe a positive effect of n-3 PUFA supplementation on bone health [52]. In that study, older women were supplemented with n-3 PUFAs at a dose of 1.2 g per day for 6 months. They also received 315 mg calcium citrate and 1000 IU cholecalciferol. Our findings are also in agreement with results from the study of Rajaram et al. [53]. That trial was performed in a group of healthy adults who received one of four diets for 8 weeks: control, enriched with n-6 PUFA, enriched with n-3 PUFA in a dose of 1 g/day or combination of n-3 and n-6 PUFA diet. There were no differences in the blood concentrations of bone turnover markers (PINP, osteocalcin, C-terminal telopeptide of type I collagen) between the four diets used. A positive effect of n-3 PUFA supplementation on bone resorption was reported by Hutchins-Wiese in a pilot study [54]. In the study, a reduction of bone resorption was observed in postmenopausal breast cancer survivors on aromatase inhibitors after 3 months of supplementation with high-dose n-3 PUFAs (4 g/day). Thus, in the current study, the dose of n-3 PUFAs may have been too low or the duration too short to influence bone turnover markers. 

The mechanism by which weight loss affects bone metabolism is complex and not fully understood. It has been suggested that bone loss may be caused by a decrease in mechanical stress in the weight-bearing skeleton during weight loss, which could influence bone turnover [55]. Another reason for increased bone resorption during caloric restriction could be reduced dietary calcium intake, which may contribute to increased bone remodeling [56]. Studies by Ricci et al. [57] showed that the increase in markers of bone resorption was associated with a higher blood concentration of parathormone (PTH) after a 6-months of energy-restricted weight-loss intervention, what could be connected with the decreased calcium intake. In the current trial, subjects were middle aged, therefore, they were not recommended calcium supplements. However, protein intake in the caloric restriction diet was close to that recommende; therefore, decreased calcium intake is unlikely. Some investigators suggest that a caloric restriction diet could decrease calcium absorption as well as that of other bone active nutrients [58]. Unfortunately, calcium concentration in the blood was not assessed in the current trial, so decreased blood calcium level after caloric restriction cannot be excluded as one of the reasons for increased bone remodeling. In this context, all subjects in the current trial had low blood vitamin D concentration, which plays an important role in intestinal calcium absorption [58]. Thus, these subjects could be vulnerable to low calcium absorption. There may also be changes in serum estrogen, glucagon-like peptide 2, growth hormone, and insulin-like growth factor I (IGF-1) or increased cortisol as well as hormones secreted by adipose tissue as previously documented by Shapses and Riedt [56]. Indeed, in the present study, we observed that changes in serum CTX-I concentration during caloric restriction diet were negatively associated with changes in BMI and serum concentration of the adipocytokine leptin but correlated positively with changes in the blood adiponectin level, which is also secreted by adipose tissue. It is well known that obese individuals have higher leptin and lower adiponectin in their blood [59,60]. The negative correlation of changes in the bone resorption marker with blood leptin during caloric restriction is confirmed in the current study. Russell et al. showed that leptin acts primarily through peripheral pathways, to enhance bone growth and maturation by increasing osteoblast number and activity [61]. Thus, in the current study, the decrease in blood leptin concentration due to weight loss during caloric restriction could result in the bone resorption marker increase. 

In our study, the serum concentration of adiponectin increased in obese subjects after 3 months of a moderately restricted diet. This result is in agreement with previous studies in mice and humans [61]. Adiponectin was reported to be a major regulator of bone mass and glucose metabolism, and it was reported by Cawthorn et al. that during caloric restriction, adipocytes from bone marrow are a significant source of adiponectin [62]. The study by Devlin et al. reported that caloric restriction in mice results in high marrow adiposity and low bone mass [63]. In turn, humans with anorexia nervosa have higher bone marrow adipose tissue, which influenced secretion of adipokines [64]. It was also documented that patients with osteoporosis exhibited increased bone marrow adipose tissue, indicating an association between bone metabolism and bone marrow adipose tissue [65].

Studies have shown that adiponectin is negatively correlated with BMI [59] in young healthy men and is connected with a decrease in bone mass in childhood [66]. Our finding showing a positive association between serum adiponectin level and blood CTX-I concentration in middle aged individuals is in agreement with a study on mice. Zhu et al. [67] performed an experiment with short-term caloric restriction in young adiponectin-deficient (Apn-/-) and control WT mice. In contrast to Apn-/- mice, control mice responded to caloric restriction with increased blood adiponectin level and decreased bone mineral density and biomechanical outcomes of limb bone and vertebrae. Unlike Apn-/- mice, control mice presented an expansion of bone marrow adipose tissue after caloric restriction and increased adiponectin expression in this adipose tissue. These findings confirm that increased adiponectin level in serum induced by short-term caloric restriction and the expansion of bone marrow adipose tissue could impair bone metabolism. However, on the other hand, there are some studies in vitro and in vivo reporting that adiponectin exerts beneficial effects on osteoblasts and bone formation [68] or that it is not associated with bone mineral density in postmenopausal women [69]. A study by Kajimura et al. [70] indicates that these opposing effects of adiponectin on bone mass are associated with the fact that this adipokine could influence two different pathways. Namely, in young individuals on a normal diet or a low-calorie diet, adiponectin blood concentration is increased by osteocalcin. In turn, adiponectin affects osteoblast proliferation to decrease osteocalcin concentration and bone mass by decreasing FoxO1 activity in a PI3 kinase-dependent manner. However, over time, in older individuals these effects are masked due to adiponectin signals in neurons of the locus coeruleus to reduce sympathetic tone and increase bone mass [70]. 

The present study has some limitations. First, there are no data concerning the physical activity of individuals. Second, we did not measure other hormones which could influence bone metabolism such as IGF-1, glucocorticoids and estrogens. Third, subjects were not tested regarding nutrients/elements participating in bone remodeling, such as blood calcium and phosphorus. 

Summing up, obesity is a condition, which requires treatment, especially a caloric-restricted diet. Short-term caloric restriction in middle-aged obese individuals results in weight loss, fat loss and some metabolic improvements. However, it seems to also result in an increase in bone turnover with increased resorption. This would be an adverse impact of weight loss. Further studies should be performed to investigate factors which could prevent bone loss during caloric restriction in obese individuals. Our findings further demonstrate that n-3 PUFA supplementation at a dose of 1.8 g per day did not prevent this adverse effect of caloric restriction on bone loss. We identified that an increase in serum adiponectin concentration was positively associated with serum bone resorption marker concentration. Potential factors which could improve bone remodeling in middle-aged obese individuals during weight loss could be the supplementation with calcium, vitamin D or n-3 PUFAs at a higher dosage (4 g/day), interventions to influence the adiponectin pathway and increased physical activity. 

## 5. Conclusions

Short-term caloric restriction in middle-aged obese individuals results in weight loss, fat loss and some metabolic improvements. However, it seems to also result in an increase in bone turnover with increased resorption. An increase in serum adiponectin after caloric restriction could promote bone resorption. N-3 PUFA supplementation at a dose of 1.8 g per day for 3 months does not appear to exert a beneficial effect on bone remodeling. Considering the beneficial effect of caloric restriction on metabolic health, further studies should be performed investigating strategies to modify the adiponectin pathway in bones which could prevent the adverse effects of weight loss on bone health.

## Figures and Tables

**Table 1:** Baseline characteristics of the subjects participating in the trial.

Parameter	All Subjects (*n* = 64)	IS + Placebo (*n* = 14)	IS + n-3 PUFA (*n* = 13)	CR + Placebo (*n* = 14)	CR + n-3 PUFA (*n* = 23)	*p*-Value *
Age (years)	41.0 ± 9.9	43 ± 11 ^1^	39 ± 5	37 ± 10	44 ± 10	0.203
Sex, female (%)	55 (*n* = 35)	50 (*n* = 7)	54 (*n* = 7)	72 (*n* = 10)	48 (*n* = 11)	>0.05
EPA in plasma PC (weight%)	2.43 ± 2.00	1.49 (1.12, 2.76) ^2^	2.42 (1.49, 4.25)	1.49 (1.11, 2.13)	1.47 (1.19, 2.67)	0.241
DHA in plasma PC (weight%)	3.78 ± 1.32	3.52 (3.03, 4.88)	4.45 (3.08, 4.74)	3.69 (2.87, 5.07)	3.36 (2.68, 4.12)	0.445
EPA in RBC membranes (weight%)	1.16 ± 0.65	1.01 (0.74, 1.54)	0.96 (0.89, 1.51)	1.050 (0.77, 1.27)	1.00 (0.83, 1.23)	0.861
DHA in RBC membranes (weight%)	4.29 ± 1.20	4.15 ± 1.30	4.34 ± 1.21	4.50 ± 1.44	4.22 ± 1.03	0.884
Omega-3 index (weight%)	5.45 ± 1.67	5.15 (4.58, 6.01)	5.73 (4.84, 6.21)	5.50 (3.97, 6.78)	5.16 (4.90, 5.69)	0.865
Weight (kg)	96.05 ± 14.29	92.20 ± 12.69	90.65 ± 16.73	100.51 ± 9.69	98.73 ± 15.33	0.168
BMI (kg/m^2^)	32.98 ± 4.01	31.56 ± 3.75	30.84 ± 3.48	35.26 ± 3.14 *	33.67 ± 4.18	0.008
Waist circumference Females (cm)	95.1 ± 13.6	96.0 (90.0, 101.5)	94.0 (88.0, 99.0)	102.0 (99.0, 106.5)	95.0 (88.0, 98.0)	0.161
Waist circumference Males (cm)	111.7 ± 9.4	105.4 ± 13.9	112.0 ± 9.0	115.0 ± 4.8	114.5 ± 5.7	0.206
WHR	0.90 ± 0.12	0.89 (0.82, 0.96)	0.95 (0.85, 0.98)	0.86 (0.82, 0.96)	0.90 (0.82, 0.99)	0.909
WHR females	0.82 ± 0.10	0.82 (0.80, 0.83)	0.83 (0.82, 0.91)	0.84 (0.80, 0.85)	0.82 (0.80, 0.83)	0.345
WHR males	0.99 ± 0.05	0.99 ± 0.06	0.98 ± 0.02	1.01 ± 0.06	1.00 ± 0.04	0.852
Adipose tissue (%)	35.16 ± 6.58	33.71 ± 7.15	31.90 ± 4.99	38.75 ± 6.88	35.85 ± 6.14	0.073
Total cholesterol (mmol/L)	5.29 ± 0.94	5.47 ± 1.20	5.36 ± 0.83	4.85 ± 0.98	5.40 ± 0.75	0.267
HDL-cholesterol (mmol/L)	1.25 ± 0.22	1.31 ± 0.23	1.20 ± 0.18	1.21 ± 0.22	1.25 ± 0.24	0.552
LDL-cholesterol (mmol/L)	3.36 ± 0.83	3.55 ± 0.90	3.58 ± 0.86	2.99 ± 0.78	3.35 ± 0.77	0.233
NEFAs (mmol/L)	0.74 ± 0.27	0.81 ± 0.32	0.65 ± 0.22	0.74 ± 0.28	0.75 ± 0.27	0.535
TGs (mmol/L)	1.54 ± 0.82	1.11 (0.87, 1.24)	1.17 (0.88, 1.70)	1.34 (1.04, 1.77)	1.71 (1.11, 2.57)	0.152
Glucose (mmol/L)	5.24 ± 0.54	4.85 (4.80, 5.63)	4.95 (4.80, 5.35)	5.08 (4.91, 5.31)	5.35 (5.05, 5.80)	0.109
Insulin (µIU/mL)	15.84 ± 8.32	14.67 ± 6.41	12.55 ± 5.16	19.84 ± 11.29	15.78 ± 8.01	0.145
HOMA-IR	3.74 ± 2.10	3.50 ± 1.86	2.84 ± 1.29	4.52 ± 2.63	3.88 ± 2.14	0.182
GIP (pg/mL)	32.54 ± 23.28	33.16 ± 13.58	22.95 ± 13.41	38.59 ± 33.40	33.52 ± 24.05	0.157
Leptin (ng/mL)	30.89 ± 22.30	26.86 ± 16.90	23.62 ± 13.61	42.12 ± 21.68	30.61 ± 27.48	0.125
Leptin females (ng/mL)	42.71 ± 23.33	36.73 ± 15.01	31.79 ± 13.20	16.70 ± 6.61	45.79 ± 33.57	0.242
Leptin males (ng/mL)	16.62 ± 8.53	17.00 ± 12.88	14.09 ± 5.75	19.56 ± 10.38	16.70 ± 6.61	0.815
Adiponectin (µg/mL)	5.81 ± 3.04	6.37 ± 4.21	6.59 ± 2.35	4.96 ± 2.48	5.56 ± 2.87	0.468
IL-6 (pg/mL)	1.47 ± 1.07	1.38 (1.01, 1.73)	0.81 (0.71, 0.99)	1.40 (1.10, 2.27)	1.08 (0.87, 1.68)	0.107
CRP (mg/L)	2.43 ± 2.14	0.90 (0.61, 3.65)	1.30 (0.63, 2.28)	3.28 (0.74, 5.04)	2.20 (0.66, 4.24)	0.331
Resistin (ng/mL)	9.49 ± 3.14	9.42 ± 3.81	9.01 ± 2.15	9.26 ± 3.08	9.96 ± 3.34	0.835
Visfatin (ng/mL)	1.16 ± 0.94	1.14 ± 0.35	0.95 ± 0.61	1.12 ± 0.67	1.33 ± 0.98	0.411
sE-selectin (pg/mL)	39.09 ± 15.30	45.13 ± 10.64	31.81 ± 12.69	37.66 ± 12.70	40.35 ± 18.92	0.170
MCP-1 (pg/mL)	352.99 ± 94.63	411.04 ± 95.39	301.49 ± 59.90	336.98 ± 65.71	356.78 ± 109.10	0.098
sVCAM-1 (ng/mL)	591.50 ± 140.37	558.32 ± 132.29	586.84 ± 123.74	581.90 ± 167.67	618.54 ± 139.05	0.657
sPECAM-1 (ng/mL)	69.17 ± 15.47	73.73 ± 15.59	69.93 ± 13.64	71.01 ± 18.41	65.08 ± 14.33	0.405
FGF-21 (pg/mL)	251.49 ± 162.01	241.04 ± 134.40	271.97 ± 218.01	257.66 ± 145.80	242.53 ± 159.93	0.886
Vitamin D (ng/mL)	14.77 ± 6.40	14.57 ± 5.70	17.15 ± 6.28	12.41 ± 5.71	14.99 ± 7.10	0.297
Osteopontin (ng/mL)	49.65 ± 11.06	49.86 ± 9.60	47.00 ± 11.60	51.50 ± 12.62	49.94 ± 11.04	0.772
PINP (ng/mL)	7.84 ± 2.10	7.06 (6.35, 8.39)	7.64 (6.73, 7.95)	8.05 (7.33, 8.42)	7.41 (6.20, 8.82)	0.334
CTX-I (ng/mL)	0.32 ± 0.13	0.29 (0.20, 0.43)	0.30 (0.22, 0.34)	0.29 (0.24, 0.32)	0.28 (0.23, 0.33)	0.647
Gla-OC (ng/mL)	10.52 ± 3.54	10.26 ± 2.66	9.81 ± 1.63	11.26 ± 3.90	10.55 ± 4.18	0.815
Glu-OC (ng/mL)	3.58 ± 2.61	3.38 ± 2.00	2.74 ± 1.61	3.46 ± 1.77	4.25 ± 3.60	0.420
% Glu-OC	24.40 ± 12.12	23.15 ± 10.26	21.17 ± 9.36	23.37 ± 9.30	27.27 ± 15.39	0.740
Total-OC (ng/mL)	14.09 ± 4.22	13.48 ± 3.47	12.52 ± 2.09	14.72 ± 4.79	14.80 ± 4.93	0.267

Significant difference between the 4 groups of subjects (*n* = 64) (one-way ANOVA or Kruskall−Wallis test for non-normally distributed variables, nominal data were analyzed by χ^2^ test), *p* < 0.05. ^1^ Mean ± SD, ^2^ Median (25–75%); all such values; * *p* < 0.05 CR+placebo group versus IS+placebo group, * *p* < 0.05 CR+placebo group versus IS+n-3 PUFA group. Abbreviations: BMI, body mass index; BP, blood pressure; CTX-I, c-terminal telopeptide of type I collagen; CR, caloric restriction; CRP, C-reactive protein; DHA, docosahexaenoic acid; EPA, eicosapentaenoic acid; FGF-21, fibroblast growth factor 21; GIP, glucose-dependent insulinotropic polypeptide; Gla-OC, carboxylated osteocalcin; Glu-OC, undercarboxylated osteocalcin; HDL, high-density lipoprotein; HOMA-IR, homeostatic model assessment; MCP-1, monocyte chemoattractant protein 1; NEFAs, nonesterified fatty acids; LDL, low-density lipoprotein; IL-6, interleukin 6; IS, isocaloric diet; OC, osteocalcin; PC, phosphatidylcholine; PINP, procollagen I n-terminal propeptide; RBC, red blood cells; sPECAM-1, soluble platelet endothelial cell adhesion molecule 1; sVCAM-1, soluble vascular cell adhesion protein 1; TGs, triglycerides; and WHR, waist-to-hip ratio.

**Table 2:** Anthropometric values and n-3 PUFAs in plasma PC and red blood cells before and after 3 months of dietary intervention in obese subjects.

Group	Time	EPA in Plasma PC (weight%)	DHA in Plasma PC (weight%)	EPA in RBC Membranes (weight%)	DHA in RBC Membranes (weight%)	Omega-3 Index (weight%)	BMI (kg/m^2^)	Adipose Tissue (%)
IS + placebo (*n* = 14)	Baseline	2.77 ± 3.14	3.91 ± 1.57	1.47 ± 1.16	4.15 ± 1.30	5.60 ± 2.30	31.56 ± 3.75	33.71 ± 7.15
	After 3 months	1.83 ± 1.04	3.59 ± 1.39	1.04 ± 0.58	4.21 ± 0.95	5.26 ± 1.41	30.87 ± 3.19	30.70 ± 7.31
IS + n-3 PUFA (*n* = 13)	Baseline	2.95 ± 1.73	4.08 ± 1.15	1.15 ± 0.43	4.34 ± 1.21	5.49 ± 1.51	30.84 ± 3.48	31.90 ± 4.99
	After 3 months	3.69 ± 1.51	6.37 ± 2.07	2.00 ± 0.55	7.12 ± 1.08	9.13 ± 1.55	31.03 ± 3.43	30.93 ± 6.93
CR + placebo (*n* = 14)	Baseline	2.03 ± 1.51	3.95 ± 1.69	1.06 ± 0.44	4.50 ± 1.44	5.56 ± 1.80	35.26 ± 3.14	38.75 ± 6.88
	After 3 months	1.57 ± 0.63	4.12 ± 1.66	0.92 ± 0.40	4.43 ± 1.40	5.35 ± 1.76	32.54 ± 4.22	37.12 ± 7.96
CR + n-3 PUFA (*n* = 23)	Baseline	2.15 ± 1.46	3.44 ± 1.01	1.06 ± 0.38	4.22 ± 1.03	5.28 ± 1.29	33.67 ± 4.18	35.85 ± 6.14
	After 3 months	3.55 ± 2.34	6.62 ± 2.11	1.97 ± 0.90	7.64 ± 1.11	9.61 ± 1.75	31.43 ± 3.94	32.25 ± 7.44
*p*-Value time x diet		0.212	<0.001	0.491	0.342	0.704	<0.001	0.050
*p*-Value time x supplementation		<0.001	<0.001	<0.001	<0.001	<0.001	0.525	0.853
*p*-Value time x diet x supplementation		0.483	0.590	0.604	0.148	0.568	0.879	0.602

Values are presented as means ± SD. Statistics: Analyses were performed with repeated measures by ANOVA. Abbreviations: BMI, body mass index; CR, caloric restriction; DHA, docosahexaenoic acid; EPA, eicosapentaenoic acid; IS, isocaloric diet; PC, phosphatidylcholine; and RBC, red blood cells.

**Table 3:** Serum lipids and glucose metabolism parameters before and after 3 month dietary intervention in obese subjects.

Group	Time	Total Cholesterol (mmol/L)	HDL- Cholesterol (mmol/L)	LDL- Cholesterol (mmol/L)	NEFAs (mmol/L)	TGs (mmol/L)	Glucose (mmol/L)	Insulin (µIU/mL)	HOMA-IR	GIP (pg/mL)
IS + placebo (*n* = 14)	Baseline	5.47 ± 1.20	1.31 ± 0.23	3.55 ± 0.90	0.81 ± 0.32	1.34 ± 0.89	5.21 ± 0.72	14.67 ± 6.41	3.50 ± 1.86	33.2 ± 13.6
	After 3 months	5.15 ± 1.01	1.32 ±0.23	3.15 ± 0.76	0.82 ± 0.28	1.51 ± 0.80	5.47 ± 0.67	15.14 ± 6.60	3.80 ± 2.10	34.9 ± 17.4
IS + n-3 PUFA (*n* = 13)	Baseline	5.36 ± 0.83	1.20 ± 0.18	3.58 ± 0.86	0.65 ± 0.22	1.29 ± 0.53	5.06 ± 0.33	12.55 ± 5.16	2.84 ± 1.29	22.9 ± 13.4
	After 3 months	5.44 ± 0.70	1.27 ± 0.17	3.56 ± 0.69	0.56 ± 0.17	1.26 ± 0.63	5.15 ± 0.36	12.52 ± 4.53	2.86 ± 1.03	19.36 ± 8.77
CR + placebo (*n* = 14)	Baseline	4.85 ± 0.98	1.21 ± 0.22	2.99 ± 0.78	0.74 ± 0.28	1.43 ± 0.54	5.09 ± 0.42	19.84 ± 11.29	4.51 ±2.63	38.6 ± 33.4
	After 3 months	4.52 ± 0.90	1.28 ± 0.34	2.67 ± 0.62	0.84 ± 0.32	1.27 ± 0.40	5.17 ± 0.35	18.05 ± 8.22	4.22 ± 2.10	34.6 ± 17.6
CR + n-3 PUFA (*n* = 23)	Baseline	5.40 ± 0.75	1.25 ± 0.24	3.35 ± 0.77	0.75 ± 0.27	1.87 ± 0.98	5.44 ± 0.53	15.78 ± 8.01	3.88 ± 2.14	33.5 ± 24.1
	After 3 months	5.09 ± 0.99	1.24 ± 0.19	3.22 ± 1.01	0.72 ± 0.23	1.52 ±0.98	5.29 ± 0.53	13.54 ± 5.57	3.25 ± 1.60	28.9 ± 24.5
*p*-Value time x diet		0.837	0.987	0.824	0.214	0.003	0.054	0.101	0.056	0.743
*p*-Value time x supplementation		0.812	0.893	0.564	0.106	0.141	0.060	0.664	0.409	0.219
*p*-Value time x diet x supplementation		0.484	0.627	0.834	0.823	0.748	0.541	0.745	0.661	0.824

Values are presented as means ± SD. Statistics: analyses were performed with repeated measures by ANOVA. Abbreviations: CR, caloric restriction; GIP, glucose-dependent insulinotropic polypeptide; HDL, high-density lipoprotein; HOMA-IR, homeostatic model assessment; NEFAs, nonesterified fatty acids; LDL, low-density lipoprotein; IS, isocaloric diet; TGs, triglycerides; and WHR, waist-to-hip ratio.

**Table 4:** Serum adipokines and markers of inflammation before and after 3 month dietary intervention.

Group	Time	Leptin (ng/mL)	Adiponectin (µg/mL)	Resistin (ng/mL)	Visfatin (ng/mL)	IL-6 (pg/mL)	CRP (mg/L)	sE-Selectin (pg/mL)	MCP-1 (pg/mL)	sPECAM-1 (ng/mL)	sVCAM-1 (ng/mL)
IS + placebo (*n* = 14)	Baseline	26.86 ± 16.90	6.37 ± 4.21	9.42 ± 3.81	1.14 ± 0.35	1.67 ± 1.02	2.12 ± 2.18	45.13 ± 10.64	411 ± 95	73.73 ± 15.59	558.3 ± 132.3
	After 3 months	24.67 ± 15.95	5.92 ± 4.20	10.04 ± 3.70	1.07 ± 0.61	1.24 ± 0.49	1.62 ± 1.29	43.12 ± 11.42	374 ± 79	65.59 ± 16.53	544.3 ± 122.4
IS + n-3 PUFA (*n* = 13)	Baseline	23.62 ± 13.61	6.59 ± 2.35	9.01 ± 2.15	0.95 ± 0.61	0.95 ± 0.44	1.51 ± 1.19	31.81 ± 12.69	302 ± 60	69.93 ± 13.63	586.8 ± 123.7
	After 3 months	21.71 ± 13.06	6.49 ± 2.36	9.08 ± 2.25	0.76 ± 0.39	0.84 ± 0.38	1.35 ± 1.02	31.05 ± 12.49	282 ± 60	63.86 ± 17.41	569.2 ± 124.2
CR + placebo (*n* = 14)	Baseline	42.12 ± 21.68	4.96 ± 2.48	9.26 ± 3.08	1.12 ± 0.67	1.75 ± 0.93	3.35 ± 2.41	37.66 ± 12.70	337 ± 66	71.01 ± 18.41	581.9 ± 167.7
	After 3 months	34.69 ± 23.31	5.43 ± 3.20	9.50 ± 3.12	0.92 ± 0.68	1.38 ± 0.72	2.36 ± 1.55	30.52 ± 10.56	324 ± 60	63.15 ± 15.12	594.5 ± 174.7
CR + n-3 PUFA (*n* = 23)	Baseline	30.61 ± 27.48	5.56 ± 2.87	9.96 ± 3.34	1.33 ± 0.98	1.49 ± 1.35	2.62 ± 2.25	40.35 ± 18.92	357 ± 109	65.08 ± 14.33	618.5 ± 139.0
	After 3 months	21.89 ± 16.51	5.87 ± 2.51	10.42 ± 3.02	1.27 ± 1.23	1.25 ± 0.66	1.52 ± 1.50	36.23 ± 16.18	337 ± 105	60.64 ± 13.52	606.9 ± 130.7
*p*-Value time x diet		0.054	0.008	0.998	0.316	0.857	0.324	0.010	0.435	0.662	0.306
*p*-Value time x supplementation		0.908	0.385	0.715	0.442	0.318	0.764	0.187	0.992	0.206	0.433
*p*-Value time x diet x suplementation		0.699	0.580	0.367	0.359	0.846	0.410	0.580	0.580	0.754	0.617

Values are presented as means ± SD. Statistics: analyses were performed with repeated-measures by ANOVA. Abbreviations: CR, caloric restriction; CRP, C-reactive protein; MCP-1, monocyte chemoattractant protein 1; IL-6, interleukin 6; IS, isocaloric diet; sPECAM-1, soluble platelet endothelial cell adhesion molecule 1; and sVCAM-1, soluble vascular cell adhesion protein 1.

**Table 5:** Vitamin D and proteins participating in bone metabolism before and after 3 month dietary intervention.

Group	Time	Vitamin D (ng/mL)	FGF-21 (pg/mL)	Osteopontin (ng/mL)	PINP (ng/mL)	CTX-I (ng/mL)	Gla-OC (ng/mL)	Glu-OC (ng/mL)	Total-OC (ng/mL)
IS + placebo (*n* = 14)	Baseline	14.57 ± 5.70	241.04 ± 134.40	49.86 ± 9.60	7.61 ± 1.85	0.32 ± 0.15	10.26 ± 2.66	3.38 ± 2.00	13.48 ± 3.48
	After 3 months	14.51 ± 4.12	206.39 ± 130.46	49.38 ± 9.66	7.64 ± 1.46	0.31 ± 0.16	10.68 ± 2.95	2.95 ± 1.37	13.63 ± 3.70
IS + n-3 PUFA (*n* = 13)	Baseline	17.15 ± 6.28	271.97 ± 218.01	47.00 ± 11.60	7.44 ± 1.23	0.32 ± 0.17	9.81 ± 1.63	2.74 ± 1.61	12.52 ± 2.09
	After 3 months	16.69 ± 6.24	239.89 ± 218.96	44.15 ± 9.37	7.73 ± 1.52	0.28 ± 0.15	10.29 ± 1.83	2.61 ± 1.59	12.71 ± 1.99
CR + placebo (*n* = 14)	Baseline	12.41 ± 5.71	257.66 ± 145.80	51.50 ± 12.62	8.38 ± 2.36	0.34 ± 0.16	11.26 ± 3.90	3.46 ± 1.77	14.72 ± 4.79
	After 3 months	13.17 ± 5.87	211.27 ± 82.91	49.81 ± 11.05	8.04 ± 1.74	0.40 ± 0.20	11.53 ± 3.57	3.35 ± 1.88	14.87 ± 4.72
CR + n-3 PUFA (*n* = 23)	Baseline	14.99 ± 7.10	242.53 ± 159.93	49.94 ± 11.04	7.87 ± 2.48	0.30 ± 0.09	10.55 ± 4.18	4.25 ± 3.60	14.80 ± 4.93
	After 3 months	15.28 ± 6.97	200.41 ± 147.24	52.84 ± 13.13	8.05 ± 1.55	0.36 ± 0.15	11.74 ± 5.23	3.88 ± 3.20	15.62 ± 5.57
*p*-Value time x diet		0.528	0.764	0.249	0.699	<0.001	0.817	0.813	0.773
*p*-Value time x supplementation		0.822	0.879	0.571	0.273	0.572	0.463	0.895	0.587
*p*-Value time x diet x suplementation		0.885	0.799	0.080	0.524	0.710	0.592	0.734	0.685

Values are presented as means ± SD. Statistics: analyses were performed with repeated-measures by ANOVA. Abbreviations: CTX-I, c-terminal telopeptide of type I collagen; CR, caloric restriction; FGF-21, fibroblast growth factor 21; Gla-OC, carboxylated osteocalcin; Glu-OC, undercarboxylated osteocalcin; IS, isocaloric diet; OC, osteocalcin; PINP, procollagen I n-terminal propeptide.

**Table 6:** Spearman rank correlation of the changes (∆) in C-terminal telopeptide of type I collagen (CTX-I) concentration and biochemical variables during a 3 months diet intervention (*n* = 64).

Parameter	∆ CTX-I (ng/mL)	
	rho	*p*
∆BMI (kg/m^2^)	−0.50	<0.001
∆Leptin (ng/mL)	−0.26	0.036
∆Adiponectin (µg/mL)	0.25	0.043

Correlations with *p* < 0.05 were considered significant. Abbreviations: BMI, body mass index; and CTX-I, C-terminal telopeptide of type I collagen.

## Data Availability

Data can be made available by contacting the corresponding author.
